# Supplementary material for: Rapid water flow triggers long-distance positive rheotaxis for thermophilic bacteria
Source: ISME J. 2025 Aug 1;19(1):wraf164. doi: 10.1093/ismejo/wraf164 (PMC12393216; doi:10.1093/ismejo/wraf164)
Supplement: 20250731_Thermus_rheotaxis_isme_sup_wraf164 [file 20250731_thermus_rheotaxis_isme_sup_wraf164.docx]

Supplementary Materials for

**Rapid water flow triggers long-distance positive rheotaxis for thermophilic bacteria**

Naoki A. Uemura^1^, Naoya Chiba^2^, Ryota Morikawa^2^, Masatada Tamakoshi^2^, Daisuke Nakane^1*^

^1^ Department of Engineering Science, The University of Electro-Communications, Tokyo, Japan. ^2^ School of Life Sciences, Tokyo University of Pharmacy and Life Sciences, Tokyo, Japan.

***Corresponding author:** Department of Engineering Science, The University of Electro-communications, 1-5-1 Chofugaoka, Chofu City, Tokyo 182-8585, Japan. Email: dice-k@uec.ac.jp

**This PDF file includes:**

Supplementary Text

Figs S1 to S13

Tables S1 to S6

Legends for Movies S1 to S11

References

**Other Supplementary Materials for this manuscript include the following:**

Movies S1 to S11

**Supplementary Text**

Construction of *pilT1*, *pilT2*, and *pilT1* *pilT2* mutants**.**

All of the mutant strains were constructed by homologous recombination using integration vectors. Knockout vectors pilT1-pyrE and pilT2-pyrE (Fig. S1AB) were constructed for insertional disruption of the *pilT1* and *pilT2* genes, respectively, using the *pyrE* gene, which codes for orotate phosphoribosyltransferase from the pyrimidine biosynthetic pathway. The *pyrE* gene cassette in p3TSDN1 [1] contains three termination codons for each different reading frame upstream of the Shine-Dalgarno sequence. The Δ*pyrE* strain AM114 [1] derived from T. thermophilus HB8 was transformed with these vectors and transformants were isolated in a synthetic minimum medium [2] without uracil. To construct the *pilT1 pilT2* double mutant, the *pyrE* gene inserted in the *pilT1::pyrE* strain was deleted by using the knockout vector pilFpilT1 (Fig. S1C) in 5-fluoroorotic acid (5-FOA) medium as described previously [3], and then the *pyrE* gene was inserted into the *pilT2* gene using pilT2-pyrE. Oligonucleotides used in this study are listed in Table S6. More detailed methods are described below.

For insertion of the pyrE gene within the *pilT1* gene (TTHA0365), a knockout vector, pilT1-pyrE (Fig. S1A), was constructed as follows. DNA fragments encoding the N- and C-terminal regions of PilT1 were amplified by PCR using the primers sets 1A-Xho/1B-Hin and 1C-Eco/1D-Bam, respectively, with T. thermophilus HB8 genomic DNA as a template. After purification, the PCR products were digested with the restriction enzyme pairs *Xho*I/*Hind*III and *Eco*RI/*Bam*HI, respectively. The digested fragments were then sequentially cloned into the corresponding sites of the plasmid p3TSDN1, in which the *Xho*I/*Hin*dIII and *Eco*RI/*Bam*HI sites were located within the multiple cloning sites at the upstream and downstream regions of the *pyrE* gene, respectively. The resulting plasmid pilT1-pyrE was used to transform Δ*pyrE* strain AM114, and the isolated transformant in MM without uracil was designated KT204. Similarly, a knockout vector for insertion of the *pyrE* gene to inactivate the *pilT2* gene (TTHA1774), pilT2-pyrE (Fig. S1B), was constructed using the two primer sets, 2A-Xho/2B-Hin and 2C-Eco/2D-Bam, and the plasmid p3TSDN1. The resulting plasmid was used to transform Δ*pyrE* strains AM114, and the isolated transformant in MM without uracil was designated KT303.

*pilT1 pilT2* double knockout strain was constructed as follows. A DNA fragment containing the C-terminal coding region of the *pilF* gene (TTHA0364) was amplified by PCR using the primers PilF-S and PilF-AS. After purification, the DNA fragment was digested with the restriction enzymes *Nde*I and *Eco*RI. The digested fragment was cloned into the corresponding sites of the plasmid pET21c. The resulting plasmid was designated pET-pilF (Fig. S1C). The *Eco*RI-*Not*I fragment of pilT1-pyrE, which includes the fragment amplified with the primer set 1C-Eco/1D-Bam and encodes the C-terminal region of PilT1, was cloned at the corresponding sites of pET-pilF. The resulting plasmid pilFpilT1 contains regions encoding the C-terminus of PilF and the C-terminus of PilT1, lacking the Shine-Dalgarno sequence of the *pilT1* gene and the regions encoding the N-terminus of PilT1. pilFpilT1 was used to transform *pilT1*::*pyrE* strain KT204 to delete the *pyrE* gene inserted within the *pilT1* gene, and 5-FOA resistant clone was isolated. Then, the resulting strain KT403 from KT204, was transformed with pilT2-pyrE for insertional inactivation of the *pilT2* gene. The isolated transformant in MM without uracil was designated KT502 derived from strains KT403.

The insertion or deletion of the *pyrE* gene in the *pilT1* and/or *pilT2* gene locus was confirmed by Southern blot analysis.

Mathematical model of bacterium moving on a planar surface using T4P filaments.

In order to reproduce the twitching motility of *T. thermophilus* on the planar surface, we propose an individual model in which the cell body is regarded as a prolate spheroid with a major radius $a$ and a short radius $b$ (Fig. S2A). There are $n$ pili, or T4P filaments, distributed on the surface near the head of the cell body. These T4P filaments are randomly distributed within a distance $a_{p}$ from the head region, along the longer axis and the circumference which is the intersection curve of a plane perpendicular to the long axis and the surface of the spheroid. The T4P filament is regarded as a linear spring with a spring constant $k$, and the extension and retraction of the T4P filaments due to the polymerization and depolymerization of pilin are modeled by changing the equilibrium length of the spring.

When a T4P filament is extended without its tip adsorbed on the planar surface, the equilibrium length of the spring increases at a constant rate $V_{e}$ (Fig. S2B). In this case, the T4P filament extends linearly in any direction within a cone with the half apex angle $\beta$ whose axis of rotation is normal to the planar surface tangent to the base of the filament. If the tip of the T4P filament contacts the planar surface, the T4P filament attached to the planar surface and turns from extension to retraction. If the equilibrium length of the spring exceeds the maximum value $d_{max}$ before it contacts the planar surface, the filament extension will also change to retraction. The retraction is achieved by shortening the equilibrium length of the spring at a constant rate $V_{r}$. If $t_{i}=0$ is the time when the tip of T4P filament $i$ is attached to the planar surface, the tension $\boldsymbol{F}_{r,i}(t_{i})$ at time $t_{i}$ is expressed as

| $\boldsymbol{F}_{r,i}\left( t_{i} \right)=k\left\{ 1-\frac{\left\vert\boldsymbol{d}_{i}\left( 0 \right) \right\vert-V_{r}\cdot t_{i}}{\left\vert\boldsymbol{d}_{i}\left( t_{i} \right) \right\vert} \right\}\boldsymbol{d}_{i}\left( t_{i} \right), \left( i=1,2,\cdots,n \right)$ | (1) |
| --- | --- |

where $\boldsymbol{d}_{i}(t_{i})$ is the direction vector from the base of the T4P filament $i$ to the attachment point at time $t_{i}$. If the magnitude of the tension $\left| \boldsymbol{F}_{r,i}(t_{i}) \right|$ is greater than $F_{max}$, the tip of the T4P filament is detached from the planar surface, and the tension then becomes zero. In addition, when the condition of $\left| \boldsymbol{d}_{i}\left( 0 \right) \right|\leq V_{r}\cdot t$ is satisfied, the tip of the T4P filament is detached from the planar surface, and the retracted filament is changed to be extended. By repeating the retraction and extension of the T4P filament with the attachment and detachment of the tip to the planar surface, the cell body translates and rotates in three-dimensional space while performing twitching motility.

Since the tips of multiple T4P filaments repeatedly attach to and detach from the planar surface asynchronously, the probability that the cell body will move far away from the planar surface is low. However, in order to avoid the cell body from slipping through the planar surface, potential energy $U_{\mathrm{LJ}}(h, \theta)$ is introduced between the cell body and the planar surface (Fig. S2C).

| $U_{\mathrm{LJ}}\left( h, \theta\right)=8a\varepsilon\left\{ \left( \frac{\sigma}{h} \right)^{10}-\left( \frac{\sigma}{h} \right)^{4}\text{cos}^{2}\theta\right\}.$ | (2) |
| --- | --- |

Equation (2) is used as the interaction potential between the liquid crystal molecules and the substrate [4], and both attractive and repulsive forces also act between the cell body and the planar surface. Here, $h$ is the minimum distance between the planar surface and the cell body, $\theta$ is the angle between the major axis vector of the cell body and the planar surface, $\varepsilon$ is the depth of the binding potential, and $\sigma$ is the distance $h$ when the potential energy becomes zero. The hydrodynamic interaction between the cell body and the planar surface is not considered to simplify the model.

The force on the prolate spheroid, which is considered as the cell body, is calculated by the sum of the tension of $n$ T4P filaments $\sum_{i=1}^{n} \boldsymbol{F}_{r,i}$ and the partial derivative of the potential energy $-\boldsymbol{\nabla}U_{\mathrm{LJ}}\left( h, \theta\right)$ from Equation (2). If the combined force is $\boldsymbol{F}$ , the torque around the principal axis of inertia of the prolate spheroid is ${\tilde{\boldsymbol{T}}}_{P}$, and the viscosity coefficient of the fluid in the tail around the cell body is $\eta$, the velocity $\boldsymbol{v}$ of the hydrodynamic center $\boldsymbol{r}=\left( x, y z \right)$ of the cell body and the angular velocity ${\tilde{\boldsymbol{\omega}}}_{P}$ of the rotation around the principal axis of inertia are expressed as follows from Stokes' resistance law[5].

| $\boldsymbol{F}=6\pi\eta a\left\{ X^{A}\boldsymbol{ee}+Y^{A}\left( I-\boldsymbol{ee} \right) \right\}\cdot\boldsymbol{v,}$ | (3) |
| --- | --- |
| ${\tilde{\boldsymbol{T}}}_{P}=8\pi\eta a^{3}\left\{ X^{C}\tilde{\boldsymbol{e}}\tilde{\boldsymbol{e}}+Y^{C}\left( I-\tilde{\boldsymbol{e}}\tilde{\boldsymbol{e}} \right) \right\}\cdot{\tilde{\boldsymbol{\omega}}}_{P}.$ | (4) |

where $\boldsymbol{e}$ is the unit vector in the direction of the major axis of the cell body, and the direction of the head is positive. Variables with tildes such as $\tilde{\boldsymbol{e}}$, ${\tilde{\boldsymbol{T}}}_{P}$ and ${\tilde{\boldsymbol{\omega}}}_{P}$ represent vectors whose coordinate axis is the principal axis of inertia of the cell body. That is, $\tilde{\boldsymbol{e}}\boldsymbol{=}\left( 1, 0, 0 \right)$. $\boldsymbol{ee}$ and $\tilde{\boldsymbol{e}}\tilde{\boldsymbol{e}}$ are the binomial products of $\boldsymbol{e}$ and $\tilde{\boldsymbol{e}}$, respectively, and $I$ is the unit tensor. $X^{A}$, $Y^{A}$, $X^{C}$ and $Y^{C}$ are the resistance tensor components of a prolate spheroid mimicking the cell body, and they are expressed as follows [5]:

| $X^{A}=\frac{8}{3}\frac{s^{3}}{\left( 1+s^{2} \right)L-2s}$, | (5-1) |
| --- | --- |
| $Y^{A}=\frac{16}{3}\frac{s^{3}}{\left( 3s^{2}-1 \right)L+2s}$, | (5-2) |
| $X^{C}=\frac{4}{3} \frac{s^{3}\left( 1-s^{2} \right)}{-\left( 1-s^{2} \right)L+2s}$, | (5-3) |
| $Y^{C}=\frac{4}{3}\frac{s^{3}\left( 2-s^{2} \right)}{\left( 1+s^{2} \right)L-2s}$. | (5-4) |

Here, $L=\ln\left\{ \left( 1+s \right)/{1-s} \right\}$ and $s={\sqrt{a^{2}-b^{2}}}/a$. Equations (3) and (4) can be solved using the Runge-Kutta method with a time step of $\Delta t=0.001$ to calculate the time variation of the center coordinate $\boldsymbol{r}$ and the unit vector $\boldsymbol{e}$ of the cell body. Note that it is not appropriate to calculate fluid flow due to external force fields.

**Figure S1.** **Construction of the integration vectors.** (A) pilT1-pyrE for inactivation of the *pilT1* gene to construct the *pilT1* strain KT204. (B) pilT2-pyrE for inactivation of the *pilT2* gene to construct the *pilT2* strain KT303, and the *pilT1 pilT2* double mutant KT502. (C) pilFpilT1 for deletion of the *pyrE* gene of KT204.

**Figure S2.** **Mathematical model of a bacterium moving with T4P filaments.** (A) A prolate spheroid is considered as a cell body. T4P filaments grow in the region of length $a_{p}$ from the tip of the head to the tail. (B) The diagram of the cycle of T4P filament extension → tip attachment → retraction → tip detachment → retraction → extension. (C) The diagram of the interaction between the cell body and the planar surface.

**
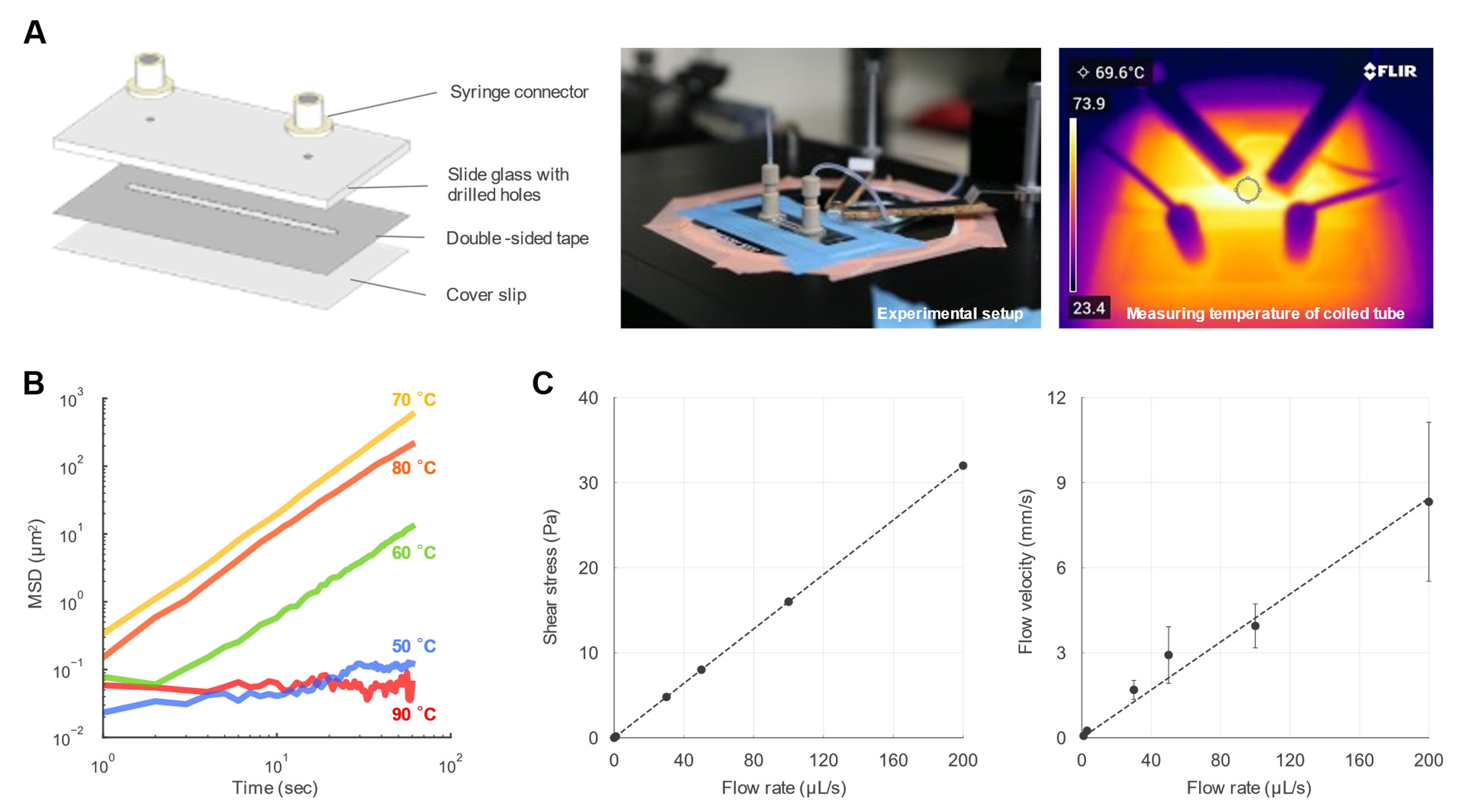
Figure S3. Temperature-controlled flow experiment.** (A) Experimental setup. *Left*: Schematic of the flow chamber assembly. *Middle*: Image of the flow chamber and tube heated on a stage heater. *Right*: Thermographic image, showing temperature distribution. The center circle temperature is presented in the upper left corner. (B) Temperature dependency of the surface motility in *T. themophilus* HB8 WT. Mean square displacement (MSD) plots of *T. thermophilus* HB8 cells at each temperature. Cells were observed in the tunnel chamber. Horizontal cells are used for data analysis (n = 40 cells). (C) Calibration of flow dynamics. *Left*: Relationship between flow rate of syringe pump and shear stress calculated based on the geometry of flow chamber. *Right*: Relationship between flow rate of syringe pump and flow velocity near the glass surface, measured from the initial velocity of detached cells (n = 8, 5, 3, 14, 5 cells at flow rates of 1, 3, 30, 50, 100, 200 µL/s). **Figure S4. Negative staining EM images of cells of *T. thermophilus* HB8 WT,** Δ***pilA*, and** Δ***pilT1pilT2* mutants.** Upper images: Originals. Lower images: Bandpass-filtered images to enhance the surface filaments. Scale bar, 500 nm.**Figure S5. Surface movement of horizontal cells.** Net displacement of horizontal cells in *T. thermophilus* HB8 WT and T4P mutants for 1 min under the nutrient-free condition without water flow. The distance of surface movement was measured from its initial to final position. Distribution, average, and SD of biological replicates are presented. n = 42 (WT), 57 (Δ*pilA*), 42 (Δ*pilT1*), 76 (Δ*pilT2*), 64 (Δ*pilT1pilT2*).

**Figure S6. Flapping motion of vertical cells.** (A) Distribution of unattached pole position relative to its attached pole during surface movement of Δ*pilT1* and Δ*pilT1pilT2* mutant cells without water flow (n = 450 from 15 cells at 1-s intervals for 30 s). (B) Distribution of unattached pole position derived from the same datasets as panel A and Fig. 2E. The solid line shows the Gaussian fitting. (C) Apparent spring constants estimated from the Gaussian fitting of y positions in panel B.

**
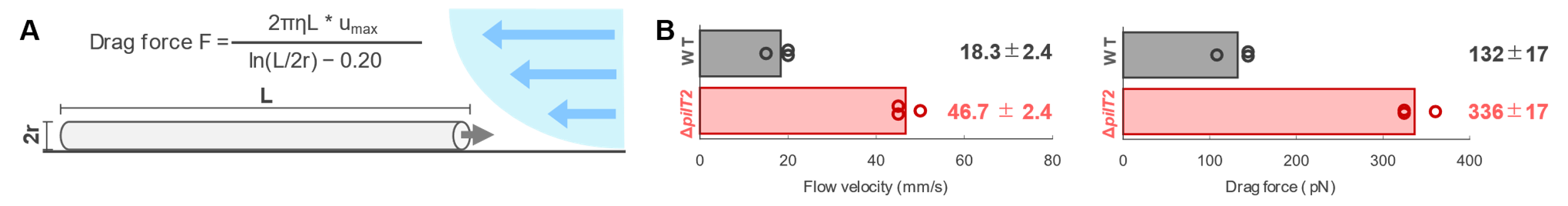
Figure S7. Maximum drag force in *T. thermophilus* HB8 WT and** Δ***pilT2*.** (A) Schematic illustration of drag force calculation. The drag coefficient was given by the cell shape assuming as a cylindrical rod with a length of 7 µm and a diameter of 0.5 µm. (B) *Left*: Maximum flow velocities where all vertical cells detached from the surface. *Right*: Maximum drag force. Drag force was estimated from the flow velocity where all vertical cells detached from the surface with the cell orientation nearly parallel to the flow direction, assuming that the cell length and width are fixed as shown in panel A. Average and SD of technical replicates are presented (n = 3 for each strain). Calibration of high flow velocity was performed separately from low flow rates.**Figure S8. Length and number of T4P filaments in vertical cells.** (A) Length distribution of T4P filament in vertical cell of *T. thermophilus* HB8 WT and Δ*pilT2* (n = 84 and 148 from 11 cells of WT and Δ*pilT2*). (B) Visible number of T4P filaments in single vertical cells of WT and Δ*pilT2* (n = 11 cells for each strain). Circles indicate biological replicates, and boxplots represent the median and 25%/75% quantile.

**
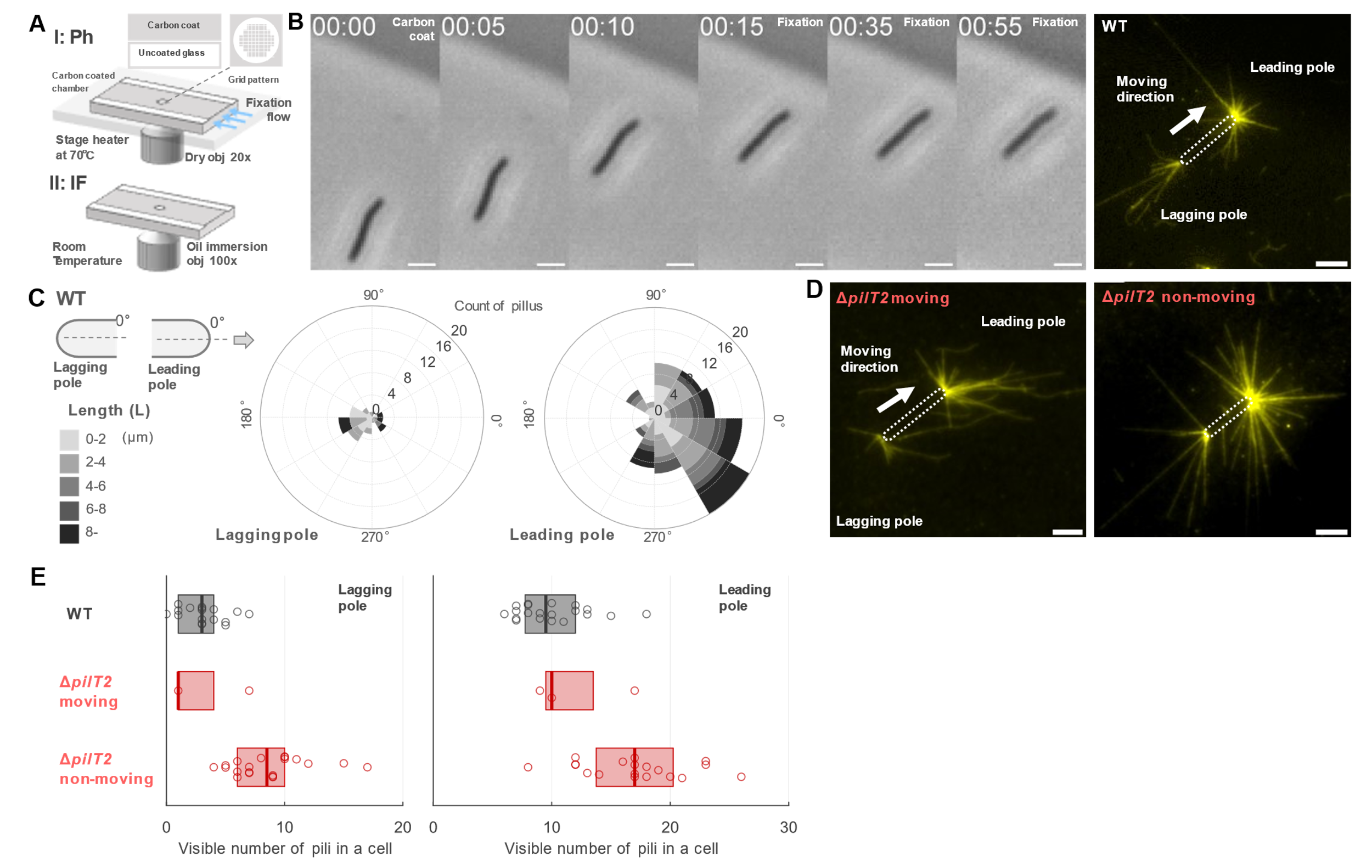
Figure S9. Visualization of T4P filaments in horizontal cells.** (A) Schematic diagram of correlation microscopy. I: Observation of cell behavior at 70°C on a stage heater under phase microscopy using a dry objective lens (low magnification). II: Immunofluorescence microscopy at RT using oil immersion objective lens (high magnification). Carbon-coated coverslips with a grid pattern were used in tunnel chambers. (B) Correlation of cell movement and T4P filaments localization. Left: Time-lapse phase-contrast images under water flow applied from the right. WT cells were chemically fixed under the nutrient-free condition at the time point of 15 s. Right: Immunofluorescence image of PilA. White dashed lines outline the cell body. Scale bar, 3 µm. (C) Length and distribution of T4P filaments. Left: Schematic of the measured parameters. θ: angle between the filament and the cell body, where the moving direction corresponds to angle zero. L: length of the T4P filaments. Right: Rose plot showing angle distribution and length of T4P filaments at the lagging (middle) and leading (right) poles (n = 11 cells). (D) Immunofluorescence image of PilA in horizontal Δ*pilT2* cells. A moving (left) and non-moving cell (right). (E) Number of visible T4P filaments in single horizontal cells. Comparisons between leading (right) and lagging poles (left) of WT (top, n = 20), moving Δ*pilT2* (middle, n = 3), and non-moving Δ*pilT2* cells (bottom, n = 20). In non-moving Δ*pilT2* cells, the pole bearing more T4P filaments was defined as the leading pole. Circles indicate biological replicates, and boxplots represent the median and 25%/75% quantile.


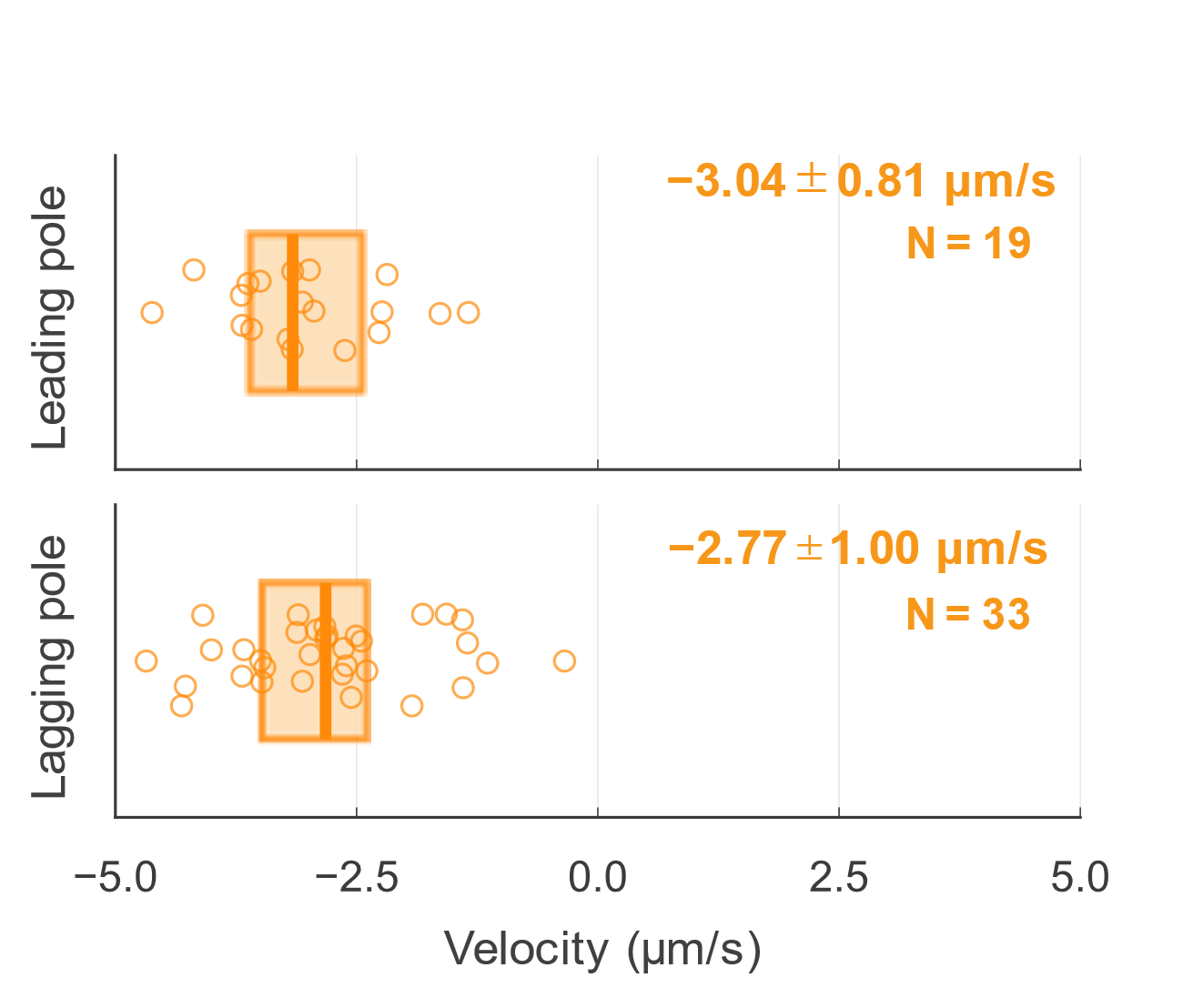


**Figure S10. Visualization of T4P dynamics in the leading and lagging cell poles.** The velocity of bead movement towards the leading and lagging poles of *T. thermophilus* HB8 WT cells. The velocity was determined by linear fitting of the bead displacement. Directional bead movements for more than 0.5 s were used for data analyses. The dataset for velocities toward the leading pole is derived from the same as that used in Fig. 4D. Bead movement towards the cell pole is defined as a negative value. Circles indicate biological replicates, and a boxplot represents the median and 25%/75% quantile (n = 33 from 9 cells of WT).

**Figure S11. Fraction of the bead dynamics.** Bead movements at the leading pole of horizontal cells in *T. thermophilus* HB8 are classified into four groups presented at the top (n = 60 events for each strain and n = 4, 12 cells for WT and Δ*pilT2*).

**Figure S12. Twitching motility in *Deinococcota*.** Mean square displacement (MSD) plots of cell movement in each strain. Cells were observed under the condition without water flow. Horizontal cells are used for the data analysis in *Thermus*. Solid and dashed lines show the average and the linear fitting of 60 s, respectively. See Table S5 for sample sizes.**Figure S13. Flapping motion in *Deinococcota*.** (A) Distribution of unattached cell pole position relative to attached cell pole (n = 450 from 15 cells at 1-s intervals for 30 s). Vertical cells of *M. ruber* 21 (left), *T. thermophilus* HB27 (middle), and *M. hydrothermalis* (right) were analyzed under the condition without water flow. (B) Distribution of unattached cell pole position along the x and y axes from the data in panel A. The black line shows the Gaussian fitting. (C) Apparent spring constants from panel B. The data set of *T. thermophilus* HB8 derived from the same experiments in Fig. S6. (D) Relationship between the apparent spring constant and the velocity of rheotaxis. The spring constants were derived from the y-axis data in panel C, while velocities of rheotaxis were data from Fig. 5C. Error bars show SD of biological replicates in the velocity of rheotaxis.

**Table S1. Strains and culture conditions**

| Strain | Medium | Temp (℃) | Reference/Source | Isolated place |
| --- | --- | --- | --- | --- |
| *Thermus thermophilus* HB8^T^ WT | NM | 70 | Tamakoshi Lab [1] | Mine-Onsen  hot spring |
| *Thermus thermophilus* HB8 Δ*pilA* | NM | 70 | Tamakoshi Lab [1] | - |
| *Thermus thermophilus* HB8 Δ*pilT1* | NM | 70 | This study | - |
| *Thermus thermophilus* HB8 Δ*pilT2* | NM | 70 | This study | - |
| *Thermus thermophilus* HB8 Δ*pilT12* | NM | 70 | This study | - |
| *Thermus thermophilus* HB27 | NM | 70 | Tamakoshi Lab [1] | Mine-Onsen  hot spring |
| *Thermus thermophilus* HB5002 | TM BROTH  JCM 273 | 70 | JCM 34562 [6] | Mine-Onsen  hot spring |
| *Thermus thermophilus* HC11 | TM BROTH | 70 | JCM 33999 [7] | Mine-Onsen  hot spring |
| *Thermus thermophilus* AK1 | TM BROTH | 70 | JCM 34718 [8] | Arima hot spring |
| *Thermus thermophilus* TMY | TM BROTH | 75 | JCM 10668 [9] | Silica scale of geothermal plant |
| *Thermus aquaticus* YT-1^T^ | CASTENHOLZ JCM 276 | 70 | JCM 10724 [10] | Hot spring in Yellow Stone National Park |
| *Thermus oshimai* SPS-17^T^ | CASTENHOLZ | 65 | JCM 11603 [11] | Hot spring in Sao Pedro do Sul |
| *Marinithermus hydrothermalis* T1^T^ | Marine broth 2216  JCM41 | 70 | JCM 11576 [12] | Deep-sea hydrothermal vent chimney |
| *Meiothermus ruber* 21^T^ | *Thermus*  NBRC 1104 | 55 | NBRC 106122 [13] | Hot spring in Kamchatka peninsula |
| *Deinococcus soli* N5^T^ | R2A  JCM 346 | 30 | JCM 19176 [14] | Rice field soil |
| *Deinococcus grandis* KS0485^T^ | NUTRIENT No2  JCM22 | 30 | JCM 6269 [15] | Eurasian carp feces |
| *Deinococcus radiodurans* R1^T^ | NUTRIENT  JCM663 | 30 | JCM 16871 [16] | Irradiated beef can |
| *Deinococcus rubellus* Ant6^T^ | R2A | 21 | JCM 31434 [17] | Antarctic fish muscle |
| *Deinococcus radiophilus* RBD^T^ | NUTRIENT | 30 | JCM 21311 [18] | Irradiated Bombay duck |

**Table S2. Composition of motility buffer**

| Strain | NaCl (mM) | pH |
| --- | --- | --- |
| *Thermus thermophilus* HB8 | 35 | 8.0 |
| *Thermus thermophilus* HB27 | 35 | 8.0 |
| *Thermus thermophilus* HB5002 | 35 | 8.0 |
| *Thermus thermophilus* HB5018 | 35 | 8.0 |
| *Thermus thermophilus* HC11 | 35 | 8.0 |
| *Thermus thermophilus* AK1 | 35 | 7.0 |
| *Thermus thermophilus* TMY | 35 | 7.5 |
| *Thermus aquaticus* YT-1 | 0 | 8.0 |
| *Thermus oshimai* SPS-17 | 35 | 8.0 |
| *Marinithermus hydrothermalis* T1 | 500 | 7.0 |
| *Meiothermus ruber* 21 | 0 | 7.5 |
| *Deinococcus soli* N5 | 0 | 7.0 |
| *Deinococcus grandis* KS0485 | 0 | 7.0 |
| *Deinococcus radiodurans* R1 | 0 | 7.0 |
| *Deinococcus rubellus* Ant6 | 0 | 7.0 |
| *Deinococcus radiophilus* RBD | 0 | 7.0 |

**Table S3. Parameters used in mathematical simulation**

| Strain | Number of pil | Retraction force limit | Retraction speed | Extension speed | Pili distribution from tip of cell |
| --- | --- | --- | --- | --- | --- |
| HB8 WT | 8 | 16.5 pN | −3.04 µm/s | 1.01 µm/s | 0.32 µm |
| HB8 Δ*pilT2* | 14 | 24.1 pN | −1.27 µm/s | 0.65 µm/s | 0.42 µm |

Number of pili was measured in Fig. S8B. Retraction force limits were estimated from drag force (Fig. S7B) divided by the number of pili . Retraction and extension speed and were measured in Fig. 4D. The distance from the tip of the cell body where the pili are distributed were measured at the leading pole of horizontal cells. (n = 12 and 10 cells for WT and Δ*pilT2*)

**Table S4. Basic parameters used in mathematical simulation**

| Cell  length $2a$ | Cell  diameter $2b$ | Spring constant  of pili $k$ | The half apex angle of the cone in which the pili can fluctuate $\beta$ | Max length  of pili | Viscosity $\eta$ | Adhesion force  of cell body $\varepsilon$ | Distance between cell body and planar surface with the potential energy zero $\sigma$ |
| --- | --- | --- | --- | --- | --- | --- | --- |
| 7.0 µm | 0.5 µm | 0.5 pN/µm | 30° | 30 µm | 0.0004 Pa∙s | 0.04 pN | 0.5 µm |

Maximum length of pili was 30 µm in Fig. S8A. Viscosity $\eta$ is that of water at 70°C.

**Table S5. Sample size for the analysis of related species**

| Strain | Twitching motility | Rheotaxis comparison | Vertical  rate |
| --- | --- | --- | --- |
| *Thermus thermophilus* HB8WT | 70 | 50 | 45 |
| *Thermus thermophilus* HB27 | 52 | 39 | 82 |
| *Thermus thermophilus* HB5002 | 48 | 31 | 49 |
| *Thermus thermophilus* HC11 | 45 | 17 | 42 |
| *Thermus thermophilus* AK1 | 57 | - | 53 |
| *Thermus thermophilus* TMY | 71 | - | 69 |
| *Thermus aquaticus* YT-1 | 79 | - | 55 |
| *Thermus oshimai* SPS-17 | 30 | 23 | 59 |
| *Marinithermus hydrothermalis* T1 | 70 | 35 | 58 |
| *Meiothermus ruber* 21 | 97 | 39 | 60 |
| *Deinococcus soli* N5 | 105 | - | 90 |
| *Deinococcus grandis* KS0485 | 137 | - | 136 |
| *Deinococcus radiodurans* R1 | 162 | - | - |
| *Deinococcus rubellus* Ant6 | 27 | - | - |
| *Deinococcus radiophilus* RBD | 92 | - | - |

**Table S6. Oligonucleotides used in this study**

| Primer name | Sequence (5’-3’) | Location |
| --- | --- | --- |
| 1A-Xho | GGCTCGAGATGGCCAAAGCCCCAGACGTG | 5’ end of the *pilT1* gene |
| 1B-Hin | GGAAGCTTCCTTGGGCTCATGGCGATCTC |
| 1C-Eco | GGGAATTCACACCAAGAGCTTCCACAAGGC | 3’ end of the *pilT1* gene |
| 1D-Bam | GGGGATCCTTACCGCCTGGCGCCCTCGG |
| 2A-Xho | GGCTCGAGATGAGCGAGGCCCAAGGCCAG | 5’ end of the *pilT2* gene |
| 2B-Hin | GGAAGCTTGGCGAGGCTTTCCATCACCTC |
| 2C-Eco | GGGAATTCTGGACACGGACAGCTTCTACAC | 3’ end of the *pilT2* gene |
| 2D-Bam | GGGGATCCTCAGTAGGTCTGCCCCGTGGC |
| PilF-S | AGGAGGCCCTGCATATGCAGAAAGGCC | 3’ end of the *pilF* gene |
| PilF-AS | GGGAATTCCTCCTTTACTCAATGGTACG |
| TTHA1221dN-S | GCGGCATATGGCTCGGAGGAACGCCAACACC | 3’ end of the *pilA* gene encoding PilA^33-122^ |
| TTHA1221dN-AS | GGGAATTCTTACTGGAAGCTAAACTGCCCGG |

The two primer sets 1A-Xho/1B-Hin and 1C-Eco/1D-Bam were used to amplify of the 5’ and 3’ ends of the *pilT1* gene, respectively, and the PCR products were used to construct pilT1-pyrE, which is the vector for insertional inactivation of the *pilT1* gene. The two primer sets 2A-Xho/2B-Hin and 2C-Eco/2D-Bam were used to amplify the 5’ and 3’ ends of the *pilT2* gene, respectively, and the PCR products were used to construct pilT2-pyrE, which is the vector for insertional inactivation of the *pilT2* gene. The primer set PilF-S/PilF-AS was used to amplify the 3’ end of the *pilF* gene, and the PCR product was used to construct pET-pilF. The primer set TTHA1221dN-S/ TTHA1221dN-AS was used to amplify the 3’ end of the *pilA* gene, and the PCR product was used to construct an expression vector for PilA^33-122^ in *E. coli*. Recognition sites for the restriction endonucleases *Xho*I, *Hin*dIII, *Eco*RI, *Bam*HI, *Nde*I, are underlined.

**Movie S1. Twitching motility of *T. thermophilu*s HB8.** WT cells were visualized in a nutrient-free buffer without water flow under a dark-field microscope for 1 min.

**Movie S2. Rheotaxis of *T. thermophilu*s HB8.** WT cells were visualized in a nutrient-free buffer under a dark-field microscope. Water flow at a speed of 40 µm/s was applied from the right side at the time of 1 min, as indicated by blue arrows. The latter half of the movie shows cell tracking automatically by TrackMate, with the mean rate of directional change, colored from grey to yellow. Yellow: positive rheotaxis of vertical cells. Grey: negative rheotaxis of horizontal cells.

**Movie S3. Long-distance migration during rheotaxis of *T. thermophilu*s HB8.** WT cells were visualized in a nutrient-free buffer under a dark-field microscope for 78 min. Water flow at a speed of 40 µm/s was applied from the right side. The yellow line indicates the overall trajectory of a typical cell exhibiting rheotaxis over 1 mm.

**Movie S4. Responses to water flow of *T. thermophilu*s HB8 in the different nutrient conditions.** *Upper*: nutrient-free buffer (same as in movie S2). *Lower*: growth medium. WT cells were visualized under a dark-field microscope for 4 min. Water flow at a speed of 40 µm/s was applied from the right side at the time of 1 min, as indicated by blue arrows.

**Movie S5. Response to water flow in T4P mutants of *T. thermophilu*s HB8.** Cells of WT, Δ*pilT1*, Δ*pilT2* and Δ*pilT1pilT2* mutants were visualized in a nutrient-free buffer under a phase-contrast microscope. White arrowheads in the initial frame indicated the vertical cells exhibiting rheotaxis. Water flow at a speed of 40 µm/s was applied from the right side at the time of 1 min, as indicated by blue arrows.

**Movie S6. Flapping motion of *T. thermophilu*s HB8.** WT and Δ*pilT2* mutant cells were visualized in a nutrient-free buffer without water flow under a phase microscope for 30 s.

**Movie S7. T4P dynamics at the leading pole of *T. thermophilu*s HB8.** WT cell in the presence of 200 nm fluorescent beads was visualized under a dark-field microscope for 10.8 s. Orange and blue triangles indicate the beads moving toward and away from the cell pole, respectively.

**Movie S8. T4P dynamics at the lagging pole of *T. thermophilu*s HB8.** WT cell in the presence of 200 nm fluorescent beads was visualized under a dark-field microscope for 4.2 s. Orange and blue triangles indicate the beads moving toward and away from the cell pole, respectively.

**Movie S9. T4P dynamics at the leading pole of *T. thermophilu*s HB8.** Δ*pilT2* mutant cell in the presence of 200 nm fluorescent beads was visualized under a dark-field microscope for 13 s. Orange and blue triangles indicate the beads moving toward and away from the cell pole, respectively.

**Movie S10. Twitching motility of *Deinococcota* bacteria.** Cells were visualized without water flow under a phase microscope for 1 min.

**Movie S11. Response to the water flow of *Deinococcota* bacteria.** Cells were visualized under a phase microscope for 1 min. Cell behavior was classified into three groups by cell shapes and orientations: Rod-shaped cells with vertical orientation, rod-shaped cells with horizontal orientation, and spherical or short rod-shaped cells. White arrowheads in the initial frame indicated the vertical cells exhibiting rheotaxis. Water flow at a speed of 40 µm/s was applied from the right side, as indicated by blue arrows.**References**

1. Tamakoshi M, Murakami A, Sugisawa M *et al.* Genomic and proteomic characterization of the large *myoviridae* bacteriophage ϕtma of the extreme thermophile *thermus thermophilus*. *Bacteriophage*. 2011;**1**:152-64 <https://doi.org/10.4161/bact.1.3.16712> , note = PMID: 22164349

2. Tanaka T, Kawano N, Oshima T. Cloning of 3-isopropylmalate dehydrogenase gene of an extreme thermophile and partial purification of the gene product. *J Biochem*. 1981;**89**:677-82 <https://doi.org/10.1093/oxfordjournals.jbchem.a133245>

3. Tamakoshi M, Yaoi T, Oshima T *et al.* An efficient gene replacement and deletion system for an extreme thermophile, *thermus thermophilus*. *FEMS Microbiol Lett*. 1999;**173**:431-37 <https://doi.org/10.1111/j.1574-6968.1999.tb13535.x>

4. Stieger T, Mazza MG, Schoen M. Diffusivity maximum in a reentrant nematic phase. *Int J Mol Sci*. 2012;**13**:7854-71 <https://doi.org/10.3390/ijms13067854>

5. Kim S, Karrila SJ. *Microhydrodynamics: Principles and selected applications*, New York: Dover Publications, 2005.

6. Miyazaki K, Moriya T, Tokito N *et al.* Complete genome sequences of *thermus thermophilus* strains hb5002 and hb5008, isolated from mine hot spring in japan. *Microbiol Resour Announc*. 2021;**10**:e00272-21 <https://doi.org/10.1128/mra.00272-21>

7. Miyazaki K. Complete genome sequencing of *thermus thermophilus* strain hc11, isolated from mine geyser in japan. *Microbiol Resour Announc*. 2019;**8**:e00873-19 <https://doi.org/10.1128/mra.00873-19>

8. Miyazaki K, Tomariguchi N, Ueno Y. Complete genome sequences of four halophilic *thermus thermophilus s*trains isolated from arima hot spring in japan. *Microbiol Resour Announc*. 2021;**10**:e00874-21 <https://doi.org/10.1128/mra.00874-21>

9. Fujino Y, Kawatsu R, Inagaki F *et al.* *Thermus thermophilus* tmy isolated from silica scale taken from a geothermal power plant. *J Appl Microbiol*. 2008;**104**:70-78 <https://doi.org/10.1111/j.1365-2672.2007.03528.x>

10. Brock TD, Freeze H. *Thermus aquaticus* gen. N. And sp. N., a nonsporulating extreme thermophile. *J Bacteriol*. 1969;**98**:289-97 <https://doi.org/10.1128/jb.98.1.289-297.1969>

11. Williams RAD, Smith KE, Welch SG *et al.* *Thermus oshimai* sp. Nov., isolated from hot springs in portugal, iceland, and the azores, and comment on the concept of a limited geographical distribution of *thermus* species. *Int J Syst Evol Microbiol*. 1996;**46**:403-08 <https://doi.org/10.1099/00207713-46-2-403>

12. Sako Y, Nakagawa S, Takai K *et al.* *Marinithermus hydrothermalis* gen. Nov., sp. Nov., a strictly aerobic, thermophilic bacterium from a deep-sea hydrothermal vent chimney. *Int J Syst Evol Microbiol*. 2003;**53**:59-65 <https://doi.org/10.1099/ijs.0.02364-0>

13. Loginova LG, Egorova LA. *Thermus ruber* obligate thermophilic bacteria in the thermal springs of kamchatka. *Mikrobiologiia*. 1975;**44**:661-5

14. Cha S, Srinivasan S, Seo T *et al.* *Deinococcus soli* sp. Nov., a gamma-radiation-resistant bacterium isolated from rice field soil. *Curr Microbiol*. 2014;**68**:777-83 <https://doi.org/10.1007/s00284-014-0542-7>

15. Oyaizu H, Stackebrandt E, Schleifer KH *et al.* A radiation-resistant rod-shaped bacterium, *deinobacter-grandis* gen-nov, sp-nov, with peptidoglycan containing ornithine. *Int J Syst Evol Microbiol*. 1987;**37**:62-67 <https://doi.org/10.1099/00207713-37-1-62>

16. Anderson AO, Nordon H, Cain RF *et al.* Studies on a radio-resistant micrococcus. I. Isolation, morphology, cultural characteristics, and resistance to gamma radiation. *Food Technol*. 1956;**10**:575-78

17. Lewis NF. Studies on a radio-resistant coccus isolated from bombay duck (*harpodon nehereus*). *J Gen Microbiol*. 1971;**66**:29-35 <https://doi.org/https://doi.org/10.1099/00221287-66-1-29>

18. Yun YS, Lee YN. Purification and some properties of superoxide dismutase from *deinococcus radiophilus* , the uv-resistant bacterium. *Extremophiles*. 2004;**8**:237-42 <https://doi.org/10.1007/s00792-004-0383-6>
